# PAMP Activity of Cerato-Platanin during Plant Interaction: An -Omic Approach

**DOI:** 10.3390/ijms17060866

**Published:** 2016-06-02

**Authors:** Simone Luti, Anna Caselli, Cosimo Taiti, Nadia Bazihizina, Cristina Gonnelli, Stefano Mancuso, Luigia Pazzagli

**Affiliations:** 1 Department of Biomedical Experimental and Clinical Sciences, Università di Firenze, viale Morgagni 50, 50134 Firenze, Italy; simone.luti@unifi.it (S.L.); anna.caselli@unifi.it (A.C.); 2 Department of Agri-Food and Environmental Science, Università di Firenze, via delle Idee 30, 50019 Sesto Fiorentino, Italy; cosimo.taiti@unifi.it (C.T.); bazihizinanadia@gmail.com (N.B.); stefano.mancuso@unifi.it (S.M.); 3 Department of Biology, Università di Firenze, via Micheli 1, 50121 Firenze, Italy; cristina.gonnelli@unifi.it

**Keywords:** cerato-platanin, expansin, PAMP, plant defense, ROS signaling, VOC accumulation

## Abstract

Cerato-platanin (CP) is the founder of a fungal protein family consisting in non-catalytic secreted proteins, which work as virulence factors and/or as elicitors of defense responses and systemic resistance, thus acting as PAMPs (pathogen-associated molecular patterns). Moreover, CP has been defined an expansin-like protein showing the ability to weaken cellulose aggregates, like the canonical plant expansins do. Here, we deepen the knowledge on CP PAMP activity by the use of a multi-disciplinary approach: proteomic analysis, VOC (volatile organic compound) measurements, and gas exchange determination. The treatment of *Arabidopsis* with CP induces a differential profile either in protein expression or in VOC emission, as well changes in photosynthetic activity. In agreement with its role of defense activator, CP treatment induces down-expression of enzymes related to primary metabolism, such as RuBisCO, triosephosphate isomerase, and ATP-synthase, and reduces the photosynthesis rate. Conversely, CP increases expression of defense-related proteins and emission of some VOCs. Interestingly, CP exposure triggered the increase in enzymes involved in GSH metabolism and redox homeostasis (glutathione *S*-transferase, thioredoxin, Cys-peroxiredoxin, catalase) and in enzymes related to the “glucosinolate-myrosinase” system, which are the premise for synthesis of defence compounds, such as camalexin and some VOCs, respectively. The presented results are in agreement with the accepted role of CP as a PAMP and greatly increase the knowledge of plant primary defences induced by a purified fungal elicitor.

## 1. Introduction

Plants have the ability to detect the presence of pathogenic microorganisms coming in contact with them by means of substances produced by microbes themselves, including several proteins [1]. Plants recognize these microbe-/pathogen-associated molecular patterns (MAMPs/PAMPs), activating a defense system that is extremely effective against the potential pathogens. The first line of defense is composed of control systems that recognize several microbe elicitors, which enable plants to shift from growth and development to defense [2,3]. Among the elicitors, essential and conserved structures for pathogen survival, a novel fungal protein family composed by Cys-rich proteins recovered either on the cell wall or in the cultural filtrate of fungi is of peculiar interest. The family has been called the Cerato-platanin family (CPF, PF07249) from the name of its founder, cerato-platanin (CP), and is now composed of more than 130 members and the number is increasing on the rise [4,5,6,7]. CP is produced by the ascomicete *Ceratocystis platani*, the causative agent of the canker stain of plane trees in Europe and North America. The protein is both abundantly secreted in the medium and present in the cell wall. As soon as the sequence of CP was deposited in the databank, other protein sequences were found with high similarity with CP so that a new fungal protein family was identified. As a consequence, the first reports on the role of CPF proteins, either in physiology of the fungus life style or in interaction with plants, have been performed on CP as representative of the family.

Regarding the primary role of the CPF proteins, recent findings hint that they are mono-domain expansin-like proteins involved in hyphal growth and development [8]. In fact, CP structure reveals a double ψβ barrel domain that shows high similarity with the D2 domain of expansins. Such a domain is conserved in all the CP structures resolved until now [5,9], and it is able to bind carbohydrates and to weaken cellulosic substrates despite lacking any enzymatic activity [10].

Regarding the role in plant interaction, CP seems to be one of the few known purified fungal proteins able to induce a systemic defense response by themselves, as it has been argued previously for bacterial flagellin and lipopolysaccharide [3]. In fact, CP is now considered to act as a PAMP in the plant interaction, being able to induce production of reactive oxygen and nitrogen species, synthesis of antimicrobial compounds, over-expression of the transcription factor WRKY 70, and synthesis of the 1–5 pathogenesis-related (PR) proteins. Moreover, CP rapidly induces mitogen-activated protein kinase (MAPK) phosphorylation, triggers salicylic acid (SA) and ethylene (ET)-signaling pathways, and causes localized resistance to the infection with *Botrytis cinerea* and *Pseudomonas syringae* [11,12]. Cerato-platanin domain-containing proteins are also detected in the secretome of *Botrytis cinerea* [13], in the soilborne *Trichoderma virens* [14], and in *Magnaporthe oryzae* [7].

Recent studies performed on pathogen infection indicate that a common response, noticed in almost all cases, is related to a decrease of photosynthetic activity, most probably due to a shift of energy resources to a general defense regulatory mechanism [15]. Simultaneously, plants contrast the pathogen infection by inducing the trancription of defense- or stress-related proteins [16]. Moreover, plants’ own versatile antioxidant systems are used to ascertain that H_2_O_2_ is maintained at low levels, letting ROS (reactive oxygen species) concentration free to act as a signaling defense inducer without excessive induction of cell damage. In fact, ROS are not simply damaging agents that induce cell death only by excessive oxidation of macromolecules, but rather they start active cell death programs [17]. Based on the statement that ROS are key signaling molecules, antioxidant enzymes and ROS scavenging gain a primary role in the fine-tuning of defense reactions: glutathione, NAD(P)H level, concentration of reduced glutathione (GSH), and over-expression of proteins from the thioredoxin superfamily concur to the redox homeostasis in the plant cell [18]. Finally, pathogen-induced ROS formation mediates the oxidation of polyunsaturated fatty acids to oxylipins, which induce the expression of genes related to the biosynthesis of secondary metabolites, such as VOCs (Volatile Organic Compounds) [19]. Among the latter molecules, a family of C6 compounds, including aldehydes, alcohols, and esters, the so-called green leaf volatiles (GVLs), are almost ubiquitously released by green plants upon abiotic (e.g., humidity, metal soil presence, temperature) and biotic stimuli [20].

To get more knowledge on the role of CP in plant interaction, a multi-disciplinary approach has been set up by the use of the so called “-omic” techniques, thus obtaining an overview of the main metabolic changes induced by a purified protein elicitor in plant interaction. In fact, until now, most of the literature deals with flg22 from bacteria and chitin, and chitosan and β-glucan from fungi as purified MAMP in interaction with hosts, but less is known about purified fungal proteins [21,22,23].

Moreover, besides the classical cases of over-expression of specific defense proteins, literature on the ensemble of proteins and secondary metabolites in plants primed by fungal elicitors is scarce [24,25]. Therefore, differentially-expressed proteins, as well as the photosynthesis rate and the emission of VOCs, were measured on *Arabidopsis* leaves treated with CP to obtain, for the first time in the field of the PAMP/plant interaction, exhaustive information on the metabolic pathways activated during defense.

## 2. Results

### 2.1. Differential Protein Expression

**Figure 1:**
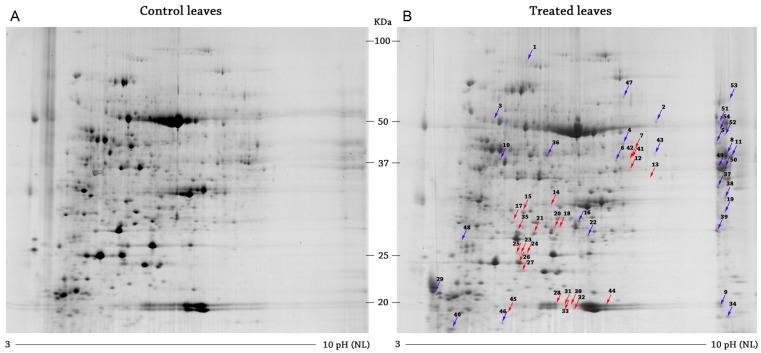
Representative reference 2-DE gels of *Arabidopsis* control (**A**) and treated (**B**). Gels were colored by colloidal Coomassie blue staining. The Progenesis SameSpot software package was used for gels analysis. The differential proteins are identified by arrows: in red, the down-expressed spots in; in blue, the over-expressed spots in cerato-platanin (CP)-treated leaves. NL, non-linear.

Proteins extracted from the CP-treated *Arabidopsis* leaves were separated by 2DE (2D-Electrophoresis and the differentially-expressed proteins were focalized between pI range 3–10 and a mass range of 12 to 100 kDa. About 1000 spots on each gel were reproducibility by Progenesis SameSpots (totallab, Newcastle, UK) (Figure 1).

Table 1 and Table 2 report results obtained by mass spectrometry, with information indicating the closest homolog proteins recovered in the database. Identification was performed after peptide mass fingerprinting, MASCOT research, and accessing the UniProt databank. Sometimes, more than one protein is present in one spot; these cases are reported as “mix score” and data interpretation takes into account that the protein with higher abundance can influence the spot quantitation and the mass spectrometry (MS) identification.

A comparison between “control” and “treated” enabled the identification of the 94 overall-affected spots: among these, 39 were downregulated and 55 were upregulated. As reported in literature, differentially-expressed proteins were defined when it was found in at least 1.5-fold abundance against control.

**Figure 2:**
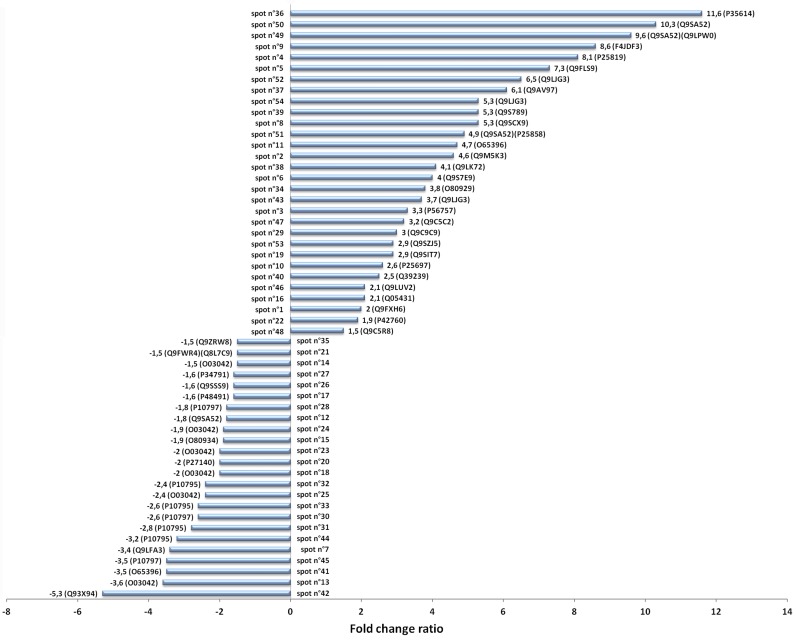
Level of expression of the identified spots. Data are negative when referred to the down-expressed proteins, while positive when referred to the over-expressed proteins. Numbers near the bar represents the fold ratio and the accession number (Uniprot databank). Bars represent the standard deviation of three replicates. Identification of each spot is provided in Table 1 and Table 2.

Of the 94 differentially-expressed spots, 54 proteins have been identified by MASCOT: 24 are down-expressed and 30 are over-expressed (Figure 2).

#### 2.1.1. Downregulated Proteins

Results show the downregulation of enzymes typically involved in primary metabolism, such as carbonic anhydrase, ribulose-1,5-bisphosphate carboxylase/oxygenase (RuBisCO), triosephosphate isomerase (TIM), and ATP-synthase-delta subunit, as shown in Table 1. In fact, despite control and treated samples being subjected to protamine to deplete most of the RuBisCO, many of the down-expressed spots contain peptides identifying the RuBisCO large and small chain (spots 13, 14, 18, 23, 24, 25 and 28, 30, 31, 32, 33, 44, 45, respectively). The chloroplast stem-loop binding protein, TIM, carbonic anhydrase, and ATP synthase delta-subunit (spots 12, 17, 20, 26, respectively), that are related to the carbon dioxide metabolism and photosynthesis, show 1.5–2-fold decrease in treated leaves. The peptidyl-prolyl cis-trans isomerase (spot 27) is also downregulated. Finally, the extracellular lipase 6 (EXL6, spot 42), involved in pollen development and growth, is largely down-expressed in our results, thus confirming the slowdown of the primary metabolism in the defense-responding leaves.

#### 2.1.2. Upregulated Proteins

Table 2 reports the over-expressed proteins identified from our gels. At a glance, the number and the fold of increase of the over-expressed proteins are twice larger than the down-expressed ones (Figure 2).

**Figure 3:**
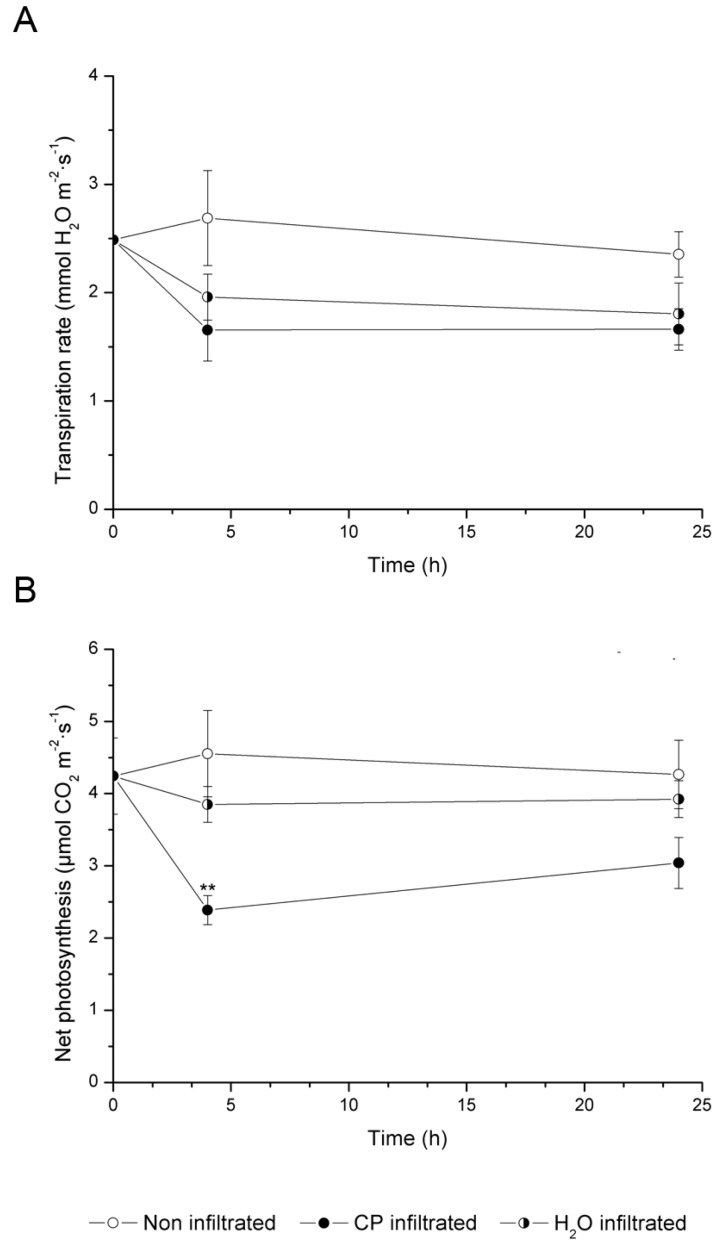
Photosynthetic rate (**A**) and transpiration rate (**B**) of *Arabidopsis thaliana* plants non-infiltrated or infiltrated with CP solution or water. Measurements were taken before any treatment and at 4 and 24 h after infiltration. Values are means ± standard deviation of three replicates (** *p* < 0.01).

One of the most over-expressed proteins is catalase-2 (spot 4), showing a concentration eight-fold higher in treated leaves than in control ones (Figure 3). The spots related to glycerol-3-phosphate dehydrogenase (spot 8) and glyceraldehyde-3-phosphate dehydrogenase (GAPDH), either the GAPA2, chloroplastic (spot 49) and GAPC1 cytosolic (spot 51) isoforms, are largely over-expressed. Finally, spot 40 has been identified as thioredoxin H4, a thiol-disulfide oxidoreductase probably involved in the redox regulation of a number of cytosolic enzymes.

Other over-expressed spots are related to proteins involved in affecting the GSH/GSSG (the ratio of reduced/oxidized forms of glutathione). An interesting result is the over-expression of glutathione *S*-transferase F6 (spot 22) and of other GSH/GSSG related-enzymes, such as 2Cys-peroxiredoxin BAS1 (spot 48) and cytosolic sulfotransferase 18 (spot 29), that are considered markers of defense as much as GSH, being involved in the cysteine pathway. Myrosinase (spot 47), an enzyme able to hydrolyze glucosinolates (GL), is also over-expressed in our study on CP-exposed *Arabidopsis* leaves. Finally, the GDSL (Gly, Asp, Ser, Leu) esterase/lipase *ESM1* (*Epithiospecifier modifier 1*) (spot 43 and 54 both identified with the Q9LJG3 accession number), belonging to the GDSL esterases/lipases family, shows a six-fold increase in treated samples.

Other over-expressed proteins are related to the glycine biosynthetic pathway and photorespiration and, therefore, ultimately related to GSH metabolism and responses to biotic and abiotic stress. In fact, the spots 2, 11, and 53 (dhydrolipoyl dehydrogenase1, amino-methyltransferase, and serine hydroxymethyl-transferase1, respectively) show a two-fold increased level in comparison to control ones. Finally, we identified some other over-expressed proteins involved in defense at different degrees. Among these an eight-fold over-expressed spot is related to a pentatricopeptide (spot 5), and the 37 and 38 spots, identified for deoxyphosphosphooctonate aldolase and for lectin-like protein (Atg16530), respectively, are involved in cell wall integrity.

Finally, the Eukaryotic peptide chain release factor (spot 36), the putative cyclic nucleotide-gated ion channel 8 (spot 1), the dirigent protein 8 (spot 9) and phosphoribulokinase, (spot 10), related to basal metabolism, are largely over-expressed in our study.

### 2.2. CO_*2*_ Assimilation and Transpiration Rate

CO_2_ assimilation and transpiration rate were determined by the use of LICOR with the aim to detect differences in photosynthesis and water between control and treated *Arabidopsis* leaves, either to add new knowledge on CP interaction with leaves or to increase information derived from proteomic analysis At all times considered, the net CO_2_ assimilation rate was negatively affected by the CP treatment (Figure 3). After four hours of treatment, net CO_2_ assimilation in CP-infiltrated plants significantly declined by approximately 50% compared with control non-infiltrated plants and H_2_O-infiltrated plants. After 24 h the difference in CO_2_ assimilation rate between CP and H_2_O-treated plants was lower, even if still significant. By contrast, no significant differences were detected between control and H_2_O-infiltrated plants to indicate that the stress induced by infiltration is smaller than that induced by CP, thus the former not affecting the validity of the experiment. The transpiration rate was negatively affected by CP treatment, but not to a significant extent. The effects on leaf water relations were less evident if compared to the decrease in photosynthetic rate, and they are largely due to the infiltration process.

### 2.3. VOC Accumulation

Volatiles as a consequence of treatment of *Arabidopsis* leaves with CP were determined to check the compounds that are induced by a purified non-catalytic fungal MAMP. Volatile organic compounds spectra from each plant were obtained by PTR-ToF-MS; peaks were present from *m*/*z* = 20 to *m*/*z* = 100. According to the procedure used by Aprea *et al.*, 2015, the data were filtered by the elimination of signals with average intensity <1 ncps.

Table 3 reports the most interesting protonated masses with the signal intensity normalized by the leaf area (expressed as normalized counts per second) for each sample. Table 4 shows the bibliographic citations in which volatile compounds have been identified by PTR-MS technologies.

Some interesting differences were obtained for the signal intensity values of the different volatiles.

All of the detectable VOCs accumulated significantly during the time of the experiment, with the only exception being the *m*/*z* 89.059, putatively identified as ethyl acetate/methyl-propanoate, in the control plants, and the *m*/*z* 27.022 and 67.054, putatively acetylene and terpene fragments, in the CP-treated plants, that did not show any remarkable changes. In CP-treated plants, in respect to control plants, the increase in VOC accumulation was significantly higher for the *m*/*z* 45.033, 49.000, 55.055, 63.027, 69.069, 71.049, 73.065, 89.59, and 101.060, which were putatively identified as acetaldehyde, methanethiol, alkyl fragment, dimethylsulfide, isoprene, 2-butenal, isobutanal/butanone, ethyl acetate/methyl-propanoate, and hexanal.

## 3. Discussion

Previous studies demonstrated the role of CP as a PAMP, being involved in primary defense responses by activating the MAPK (mitogen-activated protein kinase) signaling, ROS production, camalexin synthesis, and the metabolic pathway leading to JA and ET production [5,11,12]. To best characterize the defense responses triggered by CP and, therefore, possibly by the other proteins belonging to the CP family, an -omic approach has been used in the present work. The results obtained from proteomic data, VOC analysis, and photosynthesis measurements are in agreement with each other and shed new light on the role of CP as a PAMP. In fact, to our knowledge, it is the first time that the PAMP/plant interaction is studied at the protein and volatile level.

**Figure 4:**
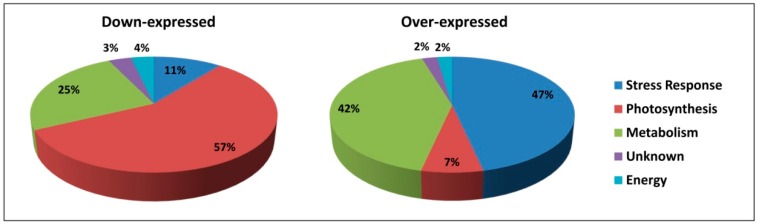
Pie-representation of differentially expressed proteins. The functional category distribution of the 54 identified proteins in Arabidopsis leaves subjected CP treatments. Data about the sub-cellular localization and biological function of each identified protein are available on Table 1 and Table 2.

Proteomic data are summarized in Figure 4, showing the pie-representation of the over- and down-expressed proteins resulting from our experiments: an increase of defense-related proteins at the expense of proteins involved in photosynthetic processes can be observed in CP-treated leaves, thus suggesting that the changing in basal carbon metabolism may increase the expression of defense-related genes, and promote the production of secondary compounds provided with antimicrobial activity.

### 3.1. CP Treatment Slows Metabolism and Negatively Affects Photosynthesis

It is widely accepted that the induction of a sink metabolism during microbe interaction is one of the shifts that leads to a conversion from source to sink tissue during plant–pathogen interactions [33]. In agreement with this general observation, CP treatment induces down-expression of RuBisCO. The significance of the other downregulated proteins here identified is in agreement with the RuBisCO result: the decrease in TIM, carbonic anhydrase, ATP synthase delta-subunit, and EXL6 testify a general slowdown of the primary metabolism. The EXL6 belongs to a large subfamily of lypolitic enzymes called GDSL esterase/lipase proteins (GELPs) which have been recovered in microbes and plants and they possess important roles in morphogenesis, development, lipid metabolism, stress responses to abiotic stimuli, and, more generally, in pathogen defense [34]. Moreover, the RuBisCO downregulation has been observed in plants infected by insects and in abiotic stress responses, and it can be considered a hallmark of a metabolic strategy to sustain fitness of plants even in a stress condition [35,36]. A reduced amount of RuBisCO has also been observed after chitosan administration to Arabidopsis leaves [37]. RuBisCO downregulation also fits with the decrease of photosynthetic activity, as shown by the substantial declines in CO_2_ assimilation after 4 h of treatment without a significant CP-induced limitation in stomatal conductance. The decrease in leaf photosynthetic rate was also in agreement with the downregulation of the peptidyl-prolyl *cis-trans* isomerase which is related to the immunophilins of the thylakoid membrane of chloroplast and has roles in regulating the assembly of photosynthetic membranes [38]. Therefore, the decrease of photosynthetic activity unavoidably brings to a metabolic shift that may have the final scope of increasing the expression of defense-related genes, thus favoring the synthesis of secondary antimicrobial metabolites as below discussed.

### 3.2. CP Treatment Increases Expression of Defense-Related Proteins and VOC Emission

Results obtained from our experiments are consistent with the general observation that the stress-induced formation of ROS is a trademark of defense activation and they enable us to increase knowledge on the activation of defenses induced by a fungal PAMP [17]. In fact, one of the largest increases in transcripts deals with proteins related to ROS scavenging and redox homeostasis: for example, catalase, which is a highly active enzyme and does not require cellular reductants, shows an eight-fold increase in treated leaves, clearly to circumvent the ROS over-production induced by CP treatment [39]. Other largely over-expressed proteins are the glycerol-3-phosphate dehydrogenase and the glyceraldehyde-3-phosphate dehydrogenase, which are known to belong to NADPH production and to oxidative stress response [40,41]. The GDSL esterase/lipase ESM1 are also over-expressed and belong to an enzyme family whose members are involved in the regulation of morphogenesis, plant development, production of secondary metabolites, and defense response [42]. Finally, other over-expressed spots identified for the pentatricopeptide, reported to be involved in response to fungus and to chitin, and for the lectin-like protein (At3g16530), involved in defense responses to oligogalacturonides [43,44].

The spots related to proteins involved in regulating the GSH/GSSG rate show the same trend and significance: the over-expression of glutathione *S*-transferase F6, 2-Cys peroxiredoxin BAS1, thioredoxin H4, and cytosolic solftransferase 18 are in agreement with the recognized role of GSH in biotic stress conditions [45,46,47]. In fact, GSH controls early signaling events, expression of stress-related genes and plant defenses under biotic stress conditions, as proved by GHS1 *Arabidopsis* mutants showing a reduction in defense responses and low production of antimicrobial compounds, such as indole glucosinolates and camalexin, in response to a wide range of pathogens [47,48]. In this respect, other enzymes involved in the Gly biosynthetic pathway (and as a consequence in the GSH biosynthesis) are over-expressed: serine hydroxymethyl-transferase1, dihydrolipoyl dehydrogenase 1 and amino-methyltransferase, to underline the central role of GSH as a scavenger of the ROS over-production during defenses. In particular, serine hydroxymethyl-transferase 1 is directly involved in controlling cell damage during the hypersensitive defense responses [48]. Monomethyltransferases are involved not only in glycine (and consequently, GSH metabolism), but also in methylation of flavonoid compounds, such as anthocyanins and naturally-occurring stilbenes, such as resveratrol [49,50]. Moreover, a low content in GSH not only affects the redox homeostasis, but also slows down the synthesis of the antimicrobial compound camalexin, that is produced in large amount during the CP/*Arabidopsis* interaction [12]. In fact, camalexin, the major phytoalexin in *Arabidopsis thaliana*, is formed by an indole ring and a thiazole ring whose sulfur derives from Cys of the GSH molecule, thus explaining the tight relation between GSH metabolism and synthesis of antimicrobial compounds [47,48,49]. Therefore, an abundance of proteins related to GSH metabolism has to be expected, and now it is demonstrated in our experimental model using a purified PAMP as a defense inducer. The contradiction in down-expression of other glutathione-S-transferases (Q9FWR4, Q8L7C9, and Q9ZRW8) is only apparent: in fact, GST-U20 (Q8L7C9) is involved in cell elongation and flowering in response to light and its down-expression is in agreement with the shifting of the metabolism in response to pathogens; on the contrary, GST-U19 (Q9ZRW8) and GST-DHAR1 (Q9FWR4) are involved in plant defense mediated by Jasmonic acid and their down-expression fits with finding that CP primes salicylic acid and ethylene signaling pathways, but not the jasmonic acid signaling [12,50,51,52,53].

Other over-expressed proteins detected in our study belong to the “glucosinolate-myrosinase” system, a unique defense mechanism typical of the *Brassicacee* family [45]. Sulfotransferase 18, myrosinase, and GDSL esterase/lipase ESM1 are involved in glucosinolate production and hydrolysis. Glucosinolates (GLs) are now considered preformed defense compounds and contribute to the protection against pathogens [45]. After cell damage, GLs are hydrolyzed by thioglucosidase enzymes (“Myrosinases”), to produce a variety of volatile products, such as thiocyanates, isothiocyanates, and nitriles, directly involved in defense, as reported by Hirschmann *et al.* [54].

The latter observation is fitting with results showing an increase of some VOCs in CP-treated leaves. Exposure to CP was able to increase the accumulation of dimethylsulfide, as it was found also in *Silene paradoxa* [55]. This VOC is enzymatically produced by plants in various environmental conditions, but its function in plant response to fungal attack is still unknown [56] The PAMP treatment induced the accumulation of another sulfur-containing compound, that of methanetiol. This molecule, together with dimethylsulfide, was found to be released by *Brassica nigra* upon herbivory and can be considered as a breakdown products of GLs, but, to the best of our knowledge, it was never associated to fungal infection [57].

Additionally, other kinds of VOCs are produced by the exposure of *Arabidopsis* plants to CP: some of them could be attributable to a PAMP-induced activation of the LOX (lipoxygenase) pathway via ROS formation, in agreement with the general observation that, due to their anti-oxidative characteristics, solubilized volatiles can also quench ROS produced during stress [20,58]. Specifically, the CP-induced upregulation of such biosynthetic pathways could have caused an accumulation of hexanal, a green leaves volatiles (GLV) C6-aldehyde that is known to be emitted by plants to counteract fungal growth [20]. In the same contest, the CP-induced accumulation of acetaldehyde could have an impact on ROS levels, as a consequence of the stress-induced activation of the LOX pathway for the biosynthesis of the GLVs [59,60]. In any case, our study is the first report about acetaldehyde production after fugal elicitation. Other CP-induced VOCs, such as the alkyl fragment, 2-butenal, isobutanal/butanone, ethyl acetate/methyl-propanoate, probably represent non-enzymatic products derived from oxidation of the polyunsaturated fatty acids upon ROS formation by CP.

Another interesting result was the CP-mediated induction of isoprene emission whereass generallys fungal infection is reported to reduce the production of such VOC [61]. Probably, the increase in the isoprene emission could be regarded as a response to the CP-mediated increase of ROS. In fact, the emission of isoprene is known to be stimulated by a wide range of environmental stresses that generate oxidative damage because of their ability in neutralizing ROS [62]. On the contrary, our VOC analysis was not able to reveal CP-mediated induction of the terpenes, as there were no signal intensities over values of *m*/*z* 100 [26]. Interestingly, signals attributable to fragments of terpenes show values of intensity lower than in control conditions or even constant over time. Therefore, even if terpenes are generally known to be produced after fungal attack and to play a role in plant defense [58], in our study the PAMP treatment seemed to downregulate their synthesis. On the other hand, it is widely accepted that environmental and biotic stress can either increase or reduce the emission rates of VOCs depending on severity, duration, and type of stress.

## 4. Materials and Methods

### 4.1. Plants and CP Treatment

*Arabidopsis thaliana* (Col-0) plants were grown in soil for five weeks in a growth chamber. Briefly, one week after germination in MS plates, *Arabidopsis* seedlings (one per container) were transferred into 120 mL containers filled with moistened soil and then closed off with a plastic lid (diameter 60 mm) perforated with five 8-mm holes by gently placing the seedlings in the central hole of the lid. Plants were then moved for five weeks in growth chambers with 12/12 h (day/night) photoperiod, 200 µmol·m^−2^·s^−1^ light intensity, 60% relative humidity, 20 °C of constant temperature and watered as required. 24 h prior to the measurements, plants were transferred in the air-conditioned room where the gas exchange were conducted. The holes in the lids were sealed with a synthetic rubber-based sealant (Terostat IX, Henkel, Düsseldorf, Germany) to suppress any potential H_2_O and CO_2_ fluxes from the soil (as confirmed by using blank pots without plants).

The CP protein used in this study was obtained from the yeast *Pichia pastoris*. The pPIC9-cp plasmid was used for transformation to permit the recovery of the protein from the cultural filtrate [63]. A single purification step by Reverse Phase-High Performance Liquid Chromatography (RP-HPLC) was needed to obtain the pure protein in high yield (60 mg from 1 L of cultured medium). Pure heterologous CP was compared with the native one both for biological activity and structure according to [64].

### 4.2. Proteomic Experiments

Six to seven leaves from five-week old plants were detached and put into a moist chamber setup in petri dishes (Figure S1). Six 10 µL drops (containing 150 µM CP or water as control) were applied on the lower surface of each leaf. Chambers were sealed and incubated under continuous light for 8 h. After incubation, drops were removed and leaves were frozen at −80 °C.

#### 4.2.1. Protein Extraction

Leaves were placed into mortar containing liquid nitrogen and pulverized with a pestle immediately after treatments. 0.4 g of leaf powder was resuspended with 2 mL of ice-cold 50 mM Tris-HCl (pH 7.5), 200 mM NaCl, 1 mM EDTA, 10 mM NaF, 2 mM sodium orthovanadate, 1 mM sodium molybdate, 10% (*v*/*v*) glycerol, 0.1% Tween 20, 1 mM phenylmethylsulfonyl fluoride, 1 mM dithiothreitol, and 1× protease inhibitor cocktail P9599 (Sigma-Aldrich, St. Louis, MO, USA). Than the samples were Ultra-Turrax (IKA, Staufen, Germany) treated for 15 s and centrifuged a 12,000× *g* for 10 min at 4 °C. The supernatant thus obtained was mixed with protamine sulfate (PS) at a final concentration of 0.05% to partially remove the RuBisCO enzyme [65]. The sample was maintain on ice for 30 min and centrifuged at 12,000× *g* for 10 min at 4 °C. Proteins in the supernatant were than subjected to methanol/chloroform precipitation. Briefly sample was added by methanol/chloroform/water (4:1:3 *v*/*v*), agitated vigorously and centrifuged for 10 min at 10,000× *g* at 4 °C. The upper layer was then removed without disturbing the interface and sample was further added of three volumes of methanol and centrifuged. The final pellet was dried, dissolved in 8M Urea, 4% (*w*/*v*) CHAPS, and 20mM DTT, and subjected to 2DE.

#### 4.2.2. 2D-Electrophoresis

2-DE replicate gels (*n* = 3) for each experimental condition, were performed using independent experiments. IEF was carried out on IPGs (pH 3–10 Non-Linear; 18 cm long IPG strips; GE Healthcare (Little Chalfont, UK)) through the Ettant IPGphor system (GE Healthcare). Strips were hydrated with 350 mL of 8 M Urea, 2% (*w*/*v*) CHAPS and 2% *v*/*v* carrier ampholyte, overnight at room temperature. Sample load, 600 µg per strip, was carried out by cup loading in the IPGphor Cup Loading Strip Holders, through the sample cup system at the anodic side of strips [66]. Subsequently, strips were placed in equilibration buffer (6 M urea, 75 mM Tris-HCl pH 8.8, 29.3% glycerol, 2% SDS) containing 2% (*w*/*v*) DTT for 15 min, and then in the same buffer with 2.5% iodoacetamide for 15 min. The second dimension was made on polyacrylamide linear gradient gels (9%–16%; 18 cm × 20 cm × 1.5 mm) at 40 mA per gel constant current. Spots were highlighted by colloidal Coomassie blue staining.

#### 4.2.3. Images Analysis

Gels image were acquired by an Epson Expression 1680 Pro image scanner. Differentially-expressed spots were selected by Progenesis SameSpot analysis (Nonlinear Dynamics, Newcastle upon Tyne, UK). Two groups (control and treated leaves) were compared with each other by the one way ANOVA analysis. All spots were pre-filtered and manually verified before applying the statistical criteria (ANOVA *p* < 0.05 and fold > 1.5). Spot intensity, instead of normalized spot volumes, was used in statistical processing.

#### 4.2.4. In-Gel Digestion and MALDI-ToF Analysis

Spots were manually cut out from gels and each sample was washed twice in 50 mM NH_4_HCO_3_/CH_3_CN 1/1 for 15 min and then dehydrated in CH_3_CN. Samples were then re-swelled in NH_4_HCO_3_ with 10 mM DTT and placed for 30 min at 56 °C; after the liquid was take out and samples were incubated in the dark for 30 min at room temperature in the same volume of 55 mM IAA in 25 mM NH_4_HCO_3_. Then, gel particles were washed twice, dried, and incubated for 30 min at 37 °C in 20 µL of 20 µg·mL^−1^ trypsin solution (Trypsin/Lys-C Mix Mass Spectrometry Grade, PROMEGA, Madison, WI, USA) in 25 mM NH_4_HCO_3_. An additional 10 µL of the buffer were added and incubated overnight at 37 °C.

The reaction was interrupted by 1% trifluoroacetic acid (TFA) and the supernatant was collected. Gel particles were then re-extracted with 1% TFA in 50% CH_3_CN. Supernatants were combined and analyzed on a MALDI-TOF/TOF mass spectrometer Ultraflex III (Bruker Daltonics, Bremen, Germany) by using Flex Control 3.0 as data acquisition software. The sample was mixed with the same volume of a saturated solution of a-cyano-4-hydroxycinnamic acid in 50% (*v*/*v*) CH_3_CN and 0.5% (*v*/*v*), and acquired in the reflectron mode over them, with a *z*-range of 860–4000, for a total of 500 shots [66].

Mass fingerprinting searching was performed in Swiss-Prot/TrEMBL databases by MASCOT (Matrix Science Ltd., London, UK, http://www.matrixscience.com) software. The taxonomy was limited to *Arabidopsis*. Alkylation of cysteine by carbamidomethylation was hired as fixed modification and a mass tolerance of 50 ppm was tolerable. The number of allowed missed cleavage sites was set to one.

### 4.3. Leaf Gas Exchange

Leaf gas exchange parameters were measured as in Bazihizina *et al.* [67], using the LI6400-XT gas exchange system analyzer equipped with a LI-6400-17 Whole Plant Arabidopsis (WPA) chamber and the 6400-18A external RGB light source, specifically planning to measure the whole plant gas exchange even on small rosette-type *Arabidopsis* plants (LICOR Inc., Lincoln, NE, USA). Four to five leaves for each plant were infiltrated with 25 µL of a 150 µM CP solution or water. All of the enclosed leaves were subjected to saturating photosynthetic photon flux density (PAR, 1000 μmol·m^−2^·s^−1^), 380 ppmv CO_2_ (achieved by fully scrubbing CO_2_ from ambient air with soda lime and replacing it with the LI-COR6400 CO_2_-injector system), 25 °C leaf temperature, and 45%–50% relative humidity. Net transpiration and photosynthetic rates were measured before any treatment and at 4 and 24 h after infiltration with water or the CP protein. Gas exchanges were also measured in non-infiltrated plants as a control. All measurements were taken on three plants from each treatment at ambient RH (60%–70%), 400 μmol·mol^−1^ CO_2_ concentration, 500 µmol·s^−1^ flow rate, 25 °C leaf chamber temperature and 200 µmol·m^−2^·s^−1^ PAR. After each measurement, leaf area was measured for all plants by the image analysis of the rosette with the Easy Leaf Area software as described by Easlon and Bloom [68].

### 4.4. Proton Transfer Reaction-Time-of-Flight-Mass Spectrometry and VOC Determination

VOC emission was detected following the method of Taiti *et al.* [55]. Briefly, shoots were isolated from the system using a synthetic rubber-based sealant (Terostat IX, Henkel, Düsseldorf, Germany) placed around the base of the stem to exclude the influence of water evaporation and ambient air. Shoots of *Arabidopsis thaliana* were uniformly sprayed with 150 µM CP solution or with milliQ-water and, immediately, plants were transferred to a glass jar (150 mL) (Figure S1). VOC accumulation was monitored by PTR-ToF-MS 8000 apparatus (Ionicon Analytik GmbH, Innsbruck, Austria) at different incubation times (0.5, 2, 4, 24 h at 25 ± 1 °C in air conditioned room). Five plants for each treatment and incubation time were evaluated, H_3_O^+^ was used as reagent ion for the proton transfer reaction. VOCs were sampled directly from the glass jar equipped on opposite sides with two holes connected with a Teflon tubes to the PTR-ToF-MS tool and to a zero-air generator, creating a dynamic headspace. A commercial zero-air generator (Peak Scientific Instruments GmbH, Frankfurt Germany) operated at 399 °C was used for the generation of VOC-free air. Mass spectra between *m*/*z* = 20–2010 was determined with ToF acquisition of 0.1 ns for each channel, the time for analyzing each plant was about 100 s. Throughout the measurement the PTR-ToF 8000 was operating in standard mode and the settings were as follows: 2.20 mbar pressure of drift tube, 60 °C temperature, 594 V drift tube voltage, 35 V of the extraction voltage at the end of the tube (Udx), corresponding to an E/N-electric field strength per gas number density-value of 130 Td (1 Td = 10–17 V·cm^−2^). The internal calibration was performed with *m*/*z* = 29.997 (NO^+^), *m*/*z* = 59.049 (C_2_H_5_O_2_^+^), and *m*/*z* = 180.937 (C_6_H_4_Cl_3_^+^) to obtain a high mass accuracy offline, following the procedure described in [69]. PTR-ToF-MS raw data were recorded by the TofDaq™ data acquisition software (Tofwerk AG, Thun, Switzerland). All spectra were corrected by the use of Poisson correction in the DAQ settings of ToFDaq configuration options. Subsequently, the TofViewer software (version 1.4.3, Ionicon Analytik, Innsbruck, Austria) was used for data post processing. Peak quantification was performed according to the duty cycle and the signals were normalized to generate normalized count per second (ncps) values. Acquisition of 60 average spectra were used for data modeling and average signal intensity was recorded for 60 s. Identification of the *m*/*z* signals was performed by assigning the mass formulas reported and through the integration of previous knowledge of the VOCs emitted by plants.

## 5. Conclusions

Topics of this paper are above the defense induction by a purified fungal protein in interaction with the model plant *Arabidopsis*. The novelty of findings is mainly due to the use of CP as a model protein either for the CPF proteins or for other non-catalytic fungal elicitors. Results obtained from different experiments are in agreement with each other and enabled a high throughput analysis of the CP/plant interaction: data obtained from proteomic, volatilomic, and gas-exchange determination largely increase the knowledge about the primary defenses induced by a purified protein elicitor. Moreover, they fit well with other studies that, however, are mainly performed with entire pathogens in interaction with the plant.

The clear fall down of the photosynthetic activity drives to a metabolic shift from source to sink in *Arabidopsis* leaves treated with CP. This is argued either by the downregulation of proteins of the primary metabolism and inhibition of CO_2_ assimilation, on one hand, and by the over-expression of enzymes involved in ROS scavenging and GSH metabolism, on the other. Moreover, results fit with some of the previously-obtained transcriptomic data. For example, the overexpression of genes corresponding to At3g16530, At1g02920, and to At2g02930, the Rossman-fold NAD(P)-binding domain containing protein, validate the overexpression of the legume lectin-like protein (Q9LK72), of the glutathione *S*-transferase (P42760) and of some dehydrogenases involved in defences (Q9M5K3, Q9SCX9, Q9LPW, P25858). Conversely, the proteins involved in regulating the GSH/GSSG rate, the enzymes involved in Gly biosynthetic pathway, and the “glucosinolate-myrosinase” system, have never been identified before in CP plant interaction. Finally, many of the data here obtained on VOCs are in agreement with proteomic results: in particular, as mentioned above, the emission of isoprene that is known to be stimulated by a wide range of environmental stresses and the overexpression of enzymes involved in synthesis of GLs that produce a variety of volatile products, related to plant defence.

## Figures and Tables

**Table 1:** Down-expressed proteins in cerato-platanin (CP)-treated leaves after 2D electrophoresis and MALDI-ToF MS analysis.

Spot No.	ID	Protein Name	MASCOT Search Results *				Theoretic pI/Mr (kDa)	Function	Localization	GO-Biological Process
			1	2	3	4				
7	Q9LFA3	Monodehydro-ascorbate reductase	16	52	214		6.4/46.6	Metabolism	Peroxisome	Oxidation-reduction process flavin adenine dinucleotide binding
12	Q9SA52		15	54	169		8.2/42.7	Metabolism	Chloroplast	Polysaccharide metabolic process Response to bacterium and abiotic stress
13	O03042	RuBisCO large chain	7	14	78		5.9/53.4	Photosynthesis	Chloroplast	Photorespiration
14	O03042	RuBisCO large chain	9	31	111		5.9/53.4	Photosynthesis	Chloroplast	Photorespiration
15	O80934	Uncharacterized protein At2g37660	15	48	204		8.4/34.9	Unknown	Chloroplast	Defense response to bacterium
17	P48491	Triosephosphate isomerase	9	47	126		5.4/27.3	Metabolism	Cytoplasm	Carbohydrate metabolic process
18	O03042	RuBisCO large chain (fragment 147–479)	14	30 (75.9)	209		5.9/53.4 (6.4/37.3)	Photosynthesis	Chloroplast	Photorespiration
20	P27140	Beta carbonic anhydrase 1	15	53	186		5.7/37.8	Photosynthesis	Chloroplast	Carbon utilization Response to bacterium Regulation of stomatal complex
21	Q9FWR4	Glutathione *S*-transferase DHAR1	15	87	225	353	5.6/23.7	Defense	mitochondrial	Response to fungus, response to Jasmonic acid
	Q8L7C9	Glutathione *S*-transferase U20	12	48	143		5.6/25.1	Metabolism/Defense	Cytoplasm/Nucleus	Regulation of growth and flowering
23	O03042	RuBisCO large chain	12	34	121		5.9/53.4	Photosynthesis	Chloroplast	Photorespiration
24	O03042	RuBisCO large chain	10	26	97		5.9/53.4	Photosynthesis	Chloroplast	Photorespiration
25	O03042	RuBisCO large chain (fragment 1–260)	9	15 (61)	84		5.9/53.4 (6.0/29.2)	Photosynthesis	Chloroplast	Photorespiration
26	Q9SSS9	ATP Synthase subunit delta	12	52	134		9.0/25.6	Energy	Chloroplast	ATP biosynthetic process photosynthetic electron transport
27	P34791	Peptidyl-prolyl cis-trans isomerase	15	66	216		8.8/28.5	Metabolism	Chloroplast	Protein folding
28	P10797	RuBisCO small chain 2B	8	35	111		7.6/20.6	Photosynthesis	Chloroplast	Photorespiration
30	P10797	RuBisCO small chain 2B	8	35	111		7.6/20.6	Photosynthesis	Chloroplast	Photorespiration
31	P10795	RuBisCO-small chain 1A	6	32	80		7.6/20.4	Photosynthesis	Chloroplast	Photorespiration
32	P10795	RuBisCO- small chain 1A	11	51	183		7.6/20.4	Photosynthesis	Chloroplast	Photorespiration
33	P10795	RuBisCO small chain 1A	15	77	245		7.6/20.4	Photosynthesis	Chloroplast	Photorespiration
35	Q9ZRW8	Glutathione S-transferase U19	7	34	95		5.8/25.6	Metabolism/Defense	Cytoplasm/Chloroplast	Response to oxidative stress; response to Jasmonic Acid
41	O65396	Aminomethyl-transferase	21	61	249		8.5/44.7	Metabolism	Mitochondrion	Glycine catabolic process
42	Q93X94	GDS-Lesterase/lipase EXL6	10	42	147		9.5/38.9	Metabolism	Secreted	Lipid catabolic process
44	P10795	RuBisCO-small chain 1A	10	42	148		7.6/20.4	Photosynthesis	Chloroplast	Photorespiration
45	P10797	RuBisCO small chain 2B	5	28	101		7.6/20.6	Photosynthesis	Chloroplast	Photorespiration

* Mascot search results: line 1: Matched Peptides; line 2: Sequence Coverage (%); Line 3: Score; Line 4: Mixed score.

**Table 2:** Over-expressed proteins in CP-treated leaves after 2D electrophoresis and MALDI-ToF MS analysis.

Spot No.	ID	Protein Name	MASCOT Search Results *				Theoretical pI/Mr (kDa)	Function	Localization	GO Biological Process
			1	2	3	4				
1	Q9FXH6	cyclic nucleotide-gated ion channel 8	11	17	81		9.15/86.7	Metabolism	Plasma membrane	Ion transport Trasmembrane potential
2	Q9M5K3	Dhydrolipoyl Dehydrogenase	16	48	230		7.0/54.2	Defense	Mitochondrion	Cell redox homeostasis Response to cadmium
3	P56757	ATP synthase subunit alpha	13	29	143		5.2/55.3	Energy	Chloroplast	ATP hydrolysis and synthesis
4	P25819	Catalase-2	20	50	275		6.6/57.2	Defense	Mitochondrion/Peroxisome	Cell redox homeostasis Response to oxidative stress
5	Q9FLS9	Pentatricopeptide repeat-At5g61800	16	35	155		8.7/56.7	Defense	Mitochondrion	Defense responses to oligogalatturonides
6	Q9S7E9	Glutamate—glyoxylate aminotransfe-rase 2	15	41	190		6.27/53.9	Metabolism	Peroxisome	Biosynthetic process l-alanine catabolic process
8	Q9SCX9	Glycerol-3-phosphate dehydrogenase	10	28	108		8.2/44.3	Metabolism/Defense	Chloroplast	Carbohydrate metabolism Glycerol 3-phosphate catabolism
9	F4JDF3	Dirigent protein 8	7	59	104		9.76/18.9	Metabolism/Defense	Apoplast	Phenylpropanoid biosynthetic process
10	P25697	Phosphoribulokinase	7	31	104		5.7/44.7	Metabolism/Defense	Chloroplast	Response to bacterium Pyrimidine salvage Pentose phosphate cycle
11	O65396	Aminomethyltransferase	14	41	174		8.5/44.7	Metabolism/Defense	Mitochondrion	Glycine catabolic process Response to cadmium
16	Q05431	l-ascorbate peroxidase 1	13	61	167		5.7/27.8	Defense	Cytoplasm	Response to oxidative stress
19	Q9SIT7	Pentatricopepti de repeat-At2g13600	5	10	61		5.6/79.1	Unknown	Mitochondrion	Mitochondrial mRNA modification
22	P42760	Glutathione S-transferase F6	13	85	205		5.8/23.4	Defense	Cell wall/Cytoplasm	Glutathione catabolic process Response to bacterium Response to abiotic stress
29	Q9C9C9	Cytosolic sulfotransferase 18	9	29	92		5.5/40.2	Metabolism/Defense	Cytoplasm	Glucosinolate biosynthetic process
34	O80929	60S ribosomal protein L36-1	5	37	72		11.7/12.7	Metabolism	Ribosome	Structural constituent of ribosome
36	P35614	Peptide chain release factor subunit 1–3	7	24	87		5.4/49.1	Metabolism	Cytoplasm	Protein biosynthesis
37	Q9AV97	2-dehydro-3-deoxyphosphooctonate aldolase	12	55	176		6.3/31.9	Metabolism	Cytoplasm	Pollen tube development and growth
38	Q9LK72	Lectin-like protein At3g16530	12	55	151		7.0/30.5	Defense	Apoplast	Defense response to fungus Response to chitin
39	Q9S789	Probable inactive cytidine deaminase 9	7	25	89		8.1/33.1	Metabolism	Cytoplasm	Cytidine deamination Pyrimidine savage
40	Q39239	Thioredoxin H4	5	34	63		5.3/13.2	Defense	Cytoplasm	Cell redox homeostasis Response to oxidative stress
43	Q9LJG3	GDSL esterase/lipase ESM1	12	47	158		7.6/44.4	Defense	Peroxisome/secreted	Responses to bacterium glucosinolate catabolism response to cold
46	Q9LUV2	Probable protein Pop3	5	73	90		5.4/12.2	Defense	Cytoplasm/Plasma membrane	Defense response to fungus Defense response to fungus
47	Q9C5C2	Myrosinase 2	25	51	284		7.1/63.3	Metabolism/Defense	Apoplast	Defense response to insect glucosinolate catabolism response to abscisic acid
48	Q9C5R8	2-Cys peroxiredoxin BAS1-like	12	51	200		5.5/29.9	Defense	Chloroplast	Cellular oxidant detoxification Responses to bacterium response to cold
49	Q9SA52	Chloroplast stem-loop binding protein of 41 kDa	17	52	172	242	8.2/42.7	Metabolism/Defense	Chloroplast	Polysaccharide metabolism Defense response to bacterium Response to abiotic stress
	Q9LPW0	Glyceraldehyde-3-phosphate dehydrogenase GAPA2	13	39	120		8.2/43.1	Metabolism	Chloroplast	Glucose metabolic process Reductive pentose phosphate cycle
50	Q9SA52	Chloroplast stem-loop binding protein	12	44	124		8.2/42.7	Metabolism	Chloroplast	Photosynthesis Polysaccharide metabolic process
51	Q9SA52	Chloroplast stem-loop binding protein	11	32	113	140	8.2/42.7	Metabolism	Chloroplast	Photosynthesis Polysaccharide metabolic process
	P25858	Glyceraldehyde-3-phosphate dehydrogenase GAPC1	7	27	65		6.6/37.0	Metabolism/Defense	Cytoplasm	Carbohydrate metabolism Response to redox state Response to abiotic stress
52	Q9LJG3	GDSL esterase/lipase ESM1	16	54	188		7.6/44.3	Defense	Secreted	Defence responses to bacterium glucosinolate catabolic process response to cold
53	Q9SZJ5	Serine hydroxymethy-transferase	18	46	187		8.1/57.5	Metabolism/Defense	Mitochondrion	Gly and Ser metabolism Hypersensitive Response tetrahydrofolate interconversion response to abiotic stress
54	Q9LJG3	GDSL esterase/lipase ESM1	6	28	95		7.6/44.3	Defense	Secreted	Responses to bacterium glucosinolate catabolism response to cold

* Mascot search results: line 1: Matched Peptides; lane 2: Sequence Coverage (%); Line 3: Score; Line 4: Mixed score.

**Table 3:** Effect of 0.5, 2, 4, and 24 h of incubation with 150 µM CP on VOC accumulation (in ncps) in *A. thaliana* plants.

Protonated Masses *m*/*z*	Time after Treatment							
	Control Plants (ncps)				CP-Treated Plants (ncps)			
	0.5 h	2 h	4 h	24 h	0.5 h	2 h	4 h	24 h
27.022	6.5 ± 0.9 aA	9.7 ± 3.4 abA	13.9 ± 1.4 bB	13.8 ± 2.7 bB	8.1 ± 1.7 aA	8.5 ± 2.2 aA	8.3 ± 2.1 aA	7.3 ± 3.5 aA
33.033	90.4 ± 33.3 aA	180.1 ± 45.4 bA	157.9 ± 45.1 bA	150.6 ± 43.1 bA	93.0 ± 23.2 aA	166.2 ± 50.4 bA	150.7 ± 24.3 bA	128.4 ± 33.1 abA
45.033	83.8 ± 5.3 aA	98.1 ± 13.2 abA	93.2 ± 16.8 abA	111.3 ± 11.4 bA	100.7 ± 12.0 aB	117.3 ± 39.2 abA	114.9 ± 37.8 abA	142.3 ± 14.6 bB
49.000	4.3 ± 1.0 aA	4.9 ± 0.7 aA	5.3 ± 0.4 aA	11.0 ± 1.6 bA	4.5 ± 0.7 aA	7.8 ± 2.3 bB	8.3 ± 1.9 bB	18.4 ± 4.1 cB
55.055	3.6 ± 0.7 aA	6.4 ± 0.3 bA	8.8 ± 3.6 cbA	11.4 ± 0.8 cA	5.0 ± 1.1 aA	7.6 ± 0.3 bB	9.6 ± 3.6 bA	15.5 ± 0.8 cB
57.033	5.0 ± 0.8 aA	7.9 ± 3.1 aA	6.2 ± 1.7 aA	7.5 ± 1.0 bA	4.3 ± 1.8 aA	4.9 ± 3.1 abA	4.4 ± 1.6 aA	7.7 ± 1.0 bA
63.027	0.7 ± 0.4 aA	0.4 ± 0.1 aA	4.1 ± 0.1 bA	4.1 ± 0.5 bA	1.8 ± 0.8 aB	0.9 ± 0.1 aB	5.0 ± 0.2 bB	3.4 ± 0.5 bA
67.054	1.9 ± 0.4 aA	4.1 ± 0.8 bB	4.9 ± 1.2 bB	4.8 ± 1.0 bB	1.9 ± 0.4 aA	2.8 ± 1.0 aA	2.1 ± 0.3 aA	2.2 ± 0.7 aA
69.069	0.8 ± 0.4 aA	2.2 ± 0.1 bA	3.1 ± 0.4 cA	4.8 ± 1.3 dA	1.2 ± 0.5 aA	2.8 ± 0.3 bB	5.0 ± 0.4 cB	10.0 ± 1.3 dB
71.049	0.8 ± 0.4 aA	2.1 ± 0.3 bA	1.8 ± 0.5 bA	2.4 ± 0.2 bA	1.9 ± 0.4 aB	2.8 ± 0.5 bA	2.7 ± 0.3 bB	4.8 ± 1.3 cB
73.065	2.3 ± 1.2 aA	3.3 ± 0.8 abA	6.0 ± 2.3 bcA	5.5 ± 1.6 bcA	4.2 ± 2.0 aA	5.2 ± 1.8 aA	7.0 ± 2.0 abA	9.5 ± 2.1 bB
75.044	18.7 ± 4.7 aA	27.1 ± 14.4 aA	88.9 ± 20.7 cA	59.9 ± 10.7 bA	23.0 ± 9.3 aA	42.0 ± 14.4 abA	72.4 ± 20.7 cA	62.3 ± 20.7 bA
81.069	1.4 ± 0.4 aB	5.8 ± 2.4 bA	5.6 ± 1.4 bB	4.6 ± 1.0 bA	0.6 ± 0.3 aA	3.5 ± 1.0 bA	1.5 ± 0.9 aA	3.7 ± 1.0 bA
89.059	3.3 ± 1.8 aA	3.3 ± 0.3 aA	3.6 ± 1.4 aA	3.7 ± 0.8 aA	4.1 ± 1.0 aA	13.0 ± 2.4 bB	15.7 ± 3.5 bB	14.6 ± 4.8 bB
93.069	1.4 ± 0.4 aA	6.7 ± 1.3 bB	6.5 ± 0.9 bA	5.7 ± 0.9 bB	1.7 ± 0.3 aA	3.6 ± 1.1 bA	5.5 ± 1.6 bA	4.1 ± 0.9 bA
101.060	0.9 ± 0.4 aA	1.4 ± 0.3 aA	4.9 ± 0.6 bA	5.1 ± 0.5 bA	0.8 ± 0.1 aA	1.8 ± 0.1 bB	4.3 ± 0.9 cA	7.6 ± 1.3 dB

Protonated masses, tentative identification, molecular formula, emission value (ncps) for treated and untreated plants with standard deviation (SD) and references of the investigated volatile compounds. Significant differences between the means appear with different letters, small for intra-treatment and capital for inter-treatment comparisons (at least *p* < 0.05).

**Table 4:** Protonated masses, tentative identification, molecular formula, and references of the investigated volatile compounds.

Protonated Masses *m*/*z*	Tentative Identification	Chemical Formulae	Reference
27.022	Acetylene	C_2_H_3_^+^	[26]
33.033	Methanol	CH_5_O^+^	[26]
45.033	Acetaldehyde	C_2_H_5_O^+^	[27]
49.000	methanethiol	CH_5_S^+^	[28]
55.055	Alkyl fragment	C_4_H_7_^+^	[29]
57.033	hexenal fragments	C_3_H_5_O^+^	[30]
63.027	Dimethylsulfide (DMS)	C_2_H_7_S^+^	[31]
67.054	Terpene fragment	C_5_H_7_^+^	[32]
69.069	Isoprene	C_5_H_9_^+^	[30]
71.049	2-butenal	C_4_H_7_O^+^	[29]
73.065	Isobutanal/Butanone	C_4_H_9_O^+^	[29]
75.044	Methyl acetate/Propanoates	C_3_H_7_O_2_^+^	[29]
81.069	Fragment/hexanal fragments	C_6_H_9_^+^	[27]
89.059	Ethyl acetate/Methyl-propanoate	C_4_H_9_O_2_^+^	[29]
93.069	terpenes alkyl fragment	C_7_H_9_^+^	[32]

## Supplementary Materials

Supplementary materials can be found at http://www.mdpi.com/1422-0067/17/6/866/s1.

## Abbreviations

2DE-SDS	Bi-Dimensional Electrophoresis-Sodium Dodecyl-Sulfate
CP	Cerato-Platanin
CPF	Cerato-platanin Family
DTT	Dithiothreitol
GLs	Glucosinolates
GLVs	Green Leaves Volatiles
GSH	Glutathione
IEF	IsoElectric Focusing
IPG	Immobilized pH Gradient
LOX	Lipoxygenase
MALDI-TOF	Matrix Assisted Laser Desorption Ionization-Time of Flight
RP-HPLC	Reverse Phase-High Performance Liquid Chromatography
M/PAMPs	Microbe-/Pathogen-Associated Molecular Patterns
ppmv	Parts Per Million by Volume
PTR-ToF-MS	Proton-Transfer-Reaction-Time of Flight-Mass Spectrometry
ROS	Reactive Oxygen Species
RuBisCO	Ribulose-1,5-bisphosphate carboxylase/oxygenase
VOCs	Volatile Organic Compounds
