# Supplementary material for: PAMP Activity of Cerato-Platanin during Plant Interaction: An -Omic Approach
Source: Int J Mol Sci. 2016 Jun 2;17(6):866. doi: 10.3390/ijms17060866 (PMC4926400; doi:10.3390/ijms17060866)
Supplement: Supplementary file 1 [file ijms-17-00866-s001.pdf]

Supplementary Materials: PAMP Activity of Cerato-Platanin during Plant Interaction: An -Omic Approach

Simone Luti, Anna Caselli, Cosimo Taiti, Nadia Bazihizina, Cristina Gonnelli, Stefano Mancuso and Luigia Pazzagli

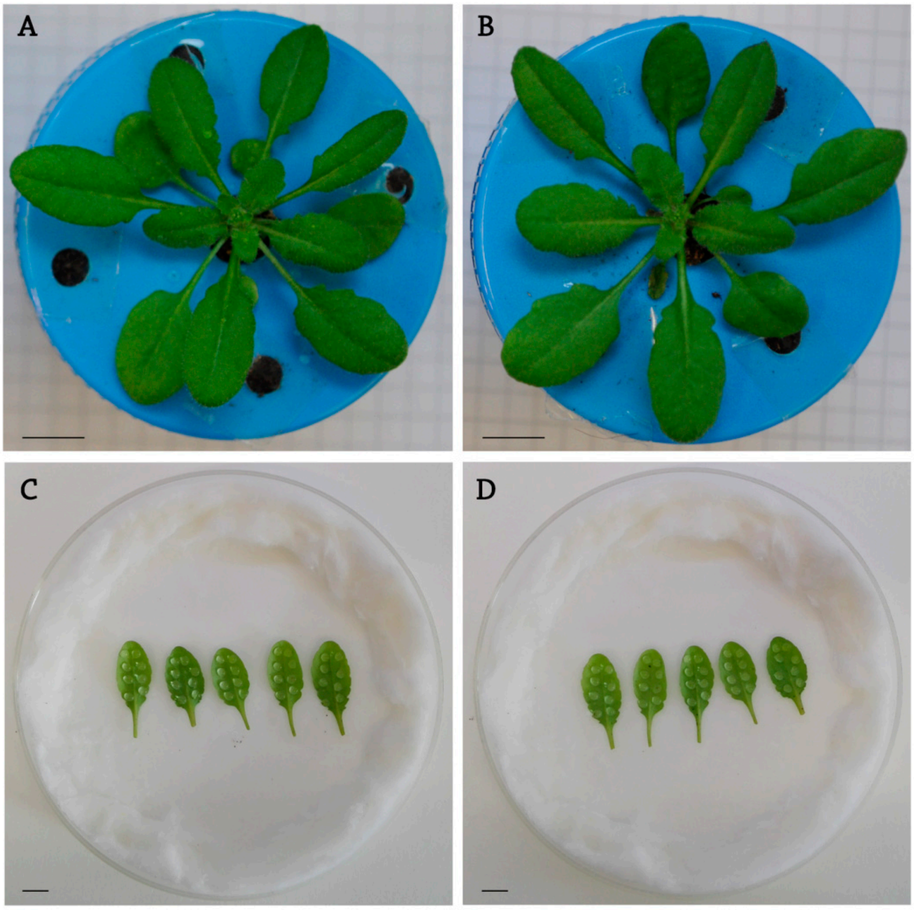

Figure S1. *Arabidopsis* plants used in gas exchange and VOCs determination experiments. (**A**) Plants treated with water (control); (**B**) plants treated with 150 μM CP (treated); (**C**) leaves added with 10 μL droplets of water (control); (**D**) leaves treated with 10 μL droplets of 150 μM cerato-platanin (CP). Bars = 1 cm.
